# Miscellaneous

**Published:** 1889-06

**Authors:** 


### MISCELLANEOUS.

STRYCHNINE TO CURE DRUNKENNESS.—A Russian physician named Portugaloff declares that strychnine is an infallible cure for drunkenness, administered in subcutaneous injection. He asserts that the experience of physicians has shown the cure to be as rapid as it is certain. The effect of the strychnine solution is to change the craving for drink into positive aversion, and this change is effected in a day. After a treatment of eight or ten days a patient may be discharged. The strychnine is administered by dissolving one grain in 200 drops of water, and injecting five drops of the solution every twenty-four hours.

A DOCTOR'S MISHAP—A somewhat amusing incident lately happened to a person who tried to bring a man out of an epileptic paroxysm by pouring cold water into his mouth and upon his neck. After a slight struggle, according to the account, the epileptic sank back apparently dead, whereupon the manipulator of the water became intensely anxious, and placed his ear at the mouth of the patient, who straightway caught the ear between his teeeth and proceeded to chew it until "its beauty had vanished." An arrest on a charge of mayhem followed the epileptic's return to consciousness; but a police justice discharged the prisoner, on the ground that he was not responsible for what he had done while in a fit.

MOSQUITOES.—The bill of a mosquito is a complex institution. It has a blunt fork at the head, and is apparently grooved. Working through the groove, and projecting from the angle of the fork, is a lance of perfect form sharpened with a fine bevel. Beside it the most perfect lance looks like a hand-saw. On either side of the lance two saws are arranged, with the points fine and sharp and the teeth well refined and keen. The backs of these saws play against the lance. When the mosquito alights with his peculiar hum, it thrusts its keen lance, and then enlarges the aperture with the two saws, which play beside the lance until the forked bill with its capillary arrangement for pumping blood can be inserted. The sawing process is what grates upon the nerves of the victim and causes him to strike wildly at the sawyer.

CEMENT FOR LEATHER.—The cement used for patching the uppers of fine shoes is generally made by dissolving gutta-percha in methylated chloroform until the mixture is about as thick as syrup. Scrape and pare clean round the hole to be covered, and thin carefully with a long chamfer the edges of the bit of leather to be applied. Only a little of the cement is needed, but the surfaces must be pressed close together. The parts will adhere firmly in a few minutes.

PINS, TWELVE DOLLARS A PAPER.—From an article entitled "Hard times in the Confederacy" in the September Century we quote the following: "In August, 1864, a private citizen's coat and vest, made of five yards of coarse homespun cloth, cost two hundred and thirty dollars exclusive of the price paid for the making. The trimmings consisted of old cravats; and for the cutting and putting together, a country tailor charged fifty dollars. It is safe to say that the private citizen looked a veritable guy in his new suit, in spite of its heavy drain upon his pocket-book.

"In January, 1865, the material for a lady's dress which before the war, would have cost ten dollars could not be bought for less than five hundred. The masculine mind is unequal to the task of guessing how great a sum might have been had for bonnets 'brought through the lines;' for in spite of patient self-sacrifice and unfaltering devotion at the bedsides of the wounded in the hospital, or in ministering to the needs of relatives and dependents at home, the Southern women of those days are credited with as keen an interest in the fashions as women everywhere in civilized lands are apt to be in times of peace. It was natural that they should be so interested, even though that interest could in the main not reach beyond theory. Without it they often would have had a charm the less, and a pang the more. Any feminine garment in the shape of cloak or bonnet or dress, which chanced to come from the North, was readily awarded its need of praise, and reproduced by sharp eyed observers, so far as the scarcity of materials would admit.

"But fashion's rules were necessarily much relaxed in the Southern Confederacy so far as practice went when even such article as pins brought through the blockade sold for twelve dollars a paper, and needles for ten, with not enough of either."

Experience has proved the CELLULOID COLLAR AND CUFF to be a prime necessity, especially in hot weather. They never wilt, are soft and pliable, and can be worn without change for months. They are the most economical goods worn—no expense for laundering. If soiled, simply wash in soap and water and they are ready again for use. Hand Sapolio is the best soap for cleaning goods. ☞ A complete assortment of these goods is sold by GEO. CLEMENT & Co., 33 East 22d St., New York.

HOW TO PREPARE CALCIMINE.—Some of our readers may wish to prepare their own calcimine, and we give these rules for the purpose: Soak one pound of white glue over night; then dissolve it in boiling water, and add twenty pounds of Paris white, diluting with water until the mixture is of the consistency of rich milk. To this any tint can be given that is desired.

*Lilac.*—Add to the calcimine two parts of Prussian blue and one of vermilion, stirring thoroughly, and taking care to avoid too high a color.

*Grey.*—Raw umber, with a trifling amount of lamp-black.

*Rose.*—Three parts of vermilion and one of red lead, added in very small quantities until a delicate shade is produced.

